# Implications of altered NAD metabolism in metabolic disorders

**DOI:** 10.1186/s12929-019-0527-8

**Published:** 2019-05-11

**Authors:** Keisuke Okabe, Keisuke Yaku, Kazuyuki Tobe, Takashi Nakagawa

**Affiliations:** 1 Department of Metabolism and Nutrition, Graduate School of Medicine and Pharmaceutical Science for Research, University of Toyama, 2630 Sugitani, Toyama, Toyama 930-0194 Japan; 2 First Department of Internal Medicine, Graduate School of Medicine and Pharmaceutical Science for Research, University of Toyama, Toyama, 930-0194 Japan; 3 Institute of Natural Medicine, University of Toyama, Toyama, 930-0194 Japan

**Keywords:** NAD, Aging, Nutritional intervention, Metabolic disease, Clinical trials

## Abstract

Nicotinamide adenine dinucleotide (NAD) is an important coenzyme that participates in various energy metabolism pathways, including glycolysis, β-oxidation, and oxidative phosphorylation. Besides, it is a required cofactor for post-translational modifications such as ADP-ribosylation and deacetylation by poly (ADP-ribose) polymerases (PARPs) and sirtuins, respectively. Thus, NAD regulates energy metabolism, DNA damage repair, gene expression, and stress response through these enzymes. Numerous studies have shown that NAD levels decrease with aging and under disturbed nutrient conditions, such as obesity. Additionally, a decline in NAD levels is closely related to the development of various metabolic disorders, including diabetes and fatty liver disease. In addition, many studies have revealed that administration of NAD precursors, such as nicotinamide mononucleotide (NMN) and nicotinamide riboside (NR), efficiently increase NAD levels in various tissues and prevent such metabolic diseases. These NAD precursors are contained in natural foods, such as cow milk, vegetables, and meats. Therefore, altered NAD metabolism can be a practical target for nutritional intervention. Recently, several human clinical trials using NAD precursors have been conducted to investigate the safety, pharmacokinetics, and efficacy against metabolic disorders such as glucose intolerance. In this review, we summarize current knowledge on the implications of NAD metabolism in metabolic diseases and discuss the outcomes of recent human clinical trials.

## Introduction

**Fig. 1:**
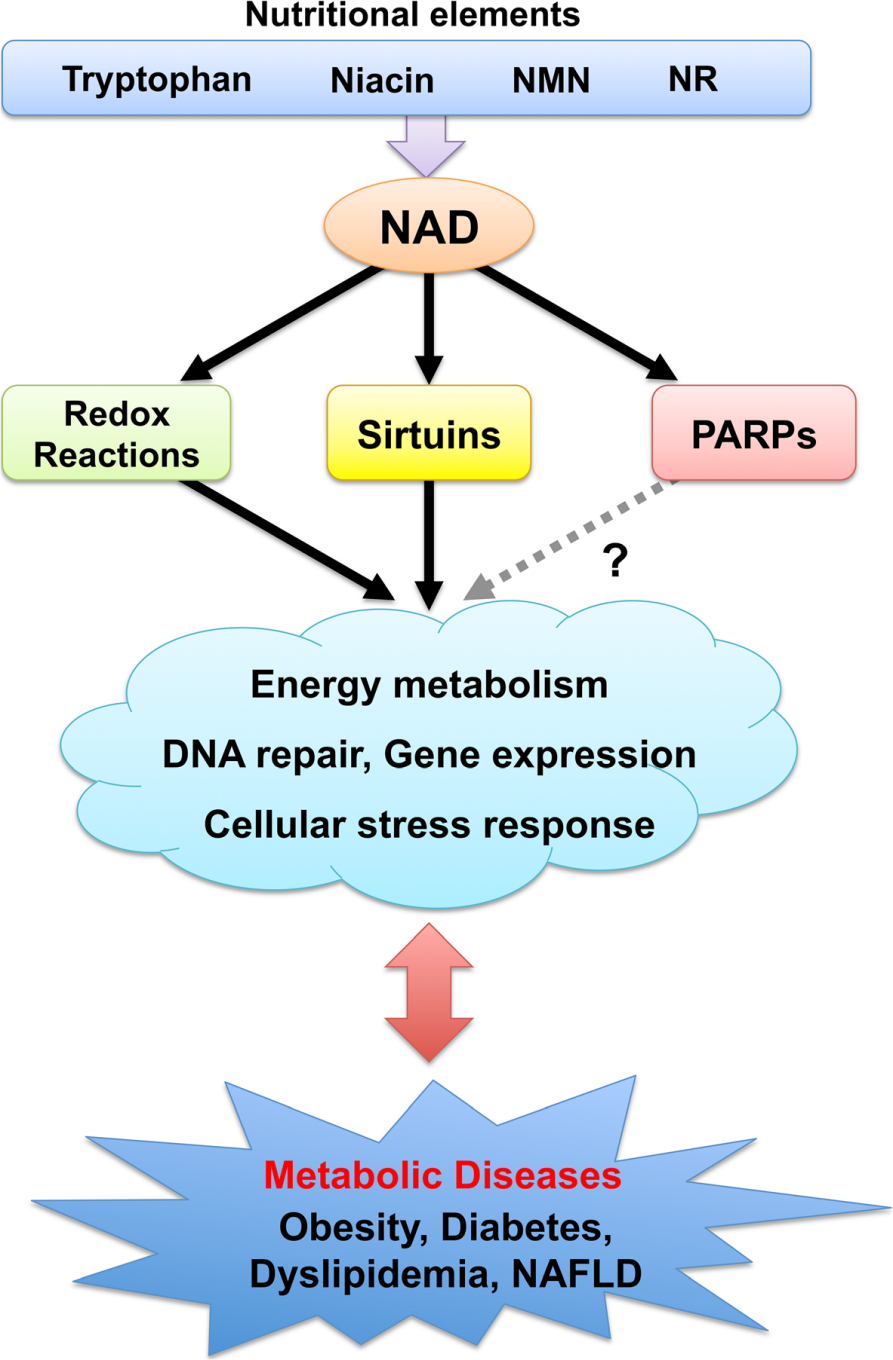
NAD metabolism has a potential protective effect against various metabolic diseases through redox reactions, sirtuins, and possibly PARPs. NAD is a co-enzyme that mediates various redox reactions in glycolysis, the TCA cycle, fatty acid oxidation, and oxidative phosphorylation. It also serves as a substrate for PARPs and sirtuins and regulates various biological pathways, including energy metabolism, gene expression, DNA repair, and cellular stress response

Metabolic syndrome is increasing worldwide and is becoming a global health concern because it is a critical risk for various life threatening diseases, including cardiovascular diseases, stroke, and cancer [1]. Its pathophysiology is based on obesity, which consequently causes diabetes, dyslipidemia, and hypertension. Development of metabolic syndrome is closely associated with nutrient status and lifestyle [2]. Excess energy intake and sedentary lifestyle cause obesity and subsequent metabolic disorders. In mammalian cells, energy-sensing pathways are important for maintaining an adequate balance between energy production and expenditure. Disturbance of these pathways results in various metabolic disorders, such as insulin resistance and fatty liver [3]. Endogenous metabolites reflect the nutrient status in cells, and their levels regulate the activity of energy-sensing molecules. For instance, adenosine monophosphate (AMP) and adenosine triphosphate (ATP) levels regulate AMP-activated protein kinase (AMPK) activity and control glucose and lipid metabolism [4]. The mammalian target of rapamycin (mTOR) senses amino acid levels and determines protein synthesis or degradation depending on nutrient availability [3]. Nicotinamide adenine dinucleotide (NAD) is also one of such energy-sensing metabolites and is an essential cofactor that mediates various biological processes, including metabolism, aging, cell death, DNA repair, and gene expression (Fig. 1) [5]. It functions as a coenzyme in various redox reactions in the major energy production pathways, such as glycolysis, tricarboxylic acid (TCA) cycle, and fatty acid oxidation [6]. NAD levels directly influence the activity of metabolic enzymes in these pathways as a coenzyme. In particular, many enzymes in the mitochondrial energy production pathway employ NAD in their redox reactions. Further, NAD acts as a substrate for poly (ADP-ribose) polymerases (PARPs) and class III NAD-dependent deacetylases (sirtuins), regulating their activities [7].

A number of studies have demonstrated that NAD levels decline with age and aberrant nutritional status, such as in obesity (Table 1) [8–23, 96]. Decreased NAD levels suppress activities of NAD (H)-dependent enzymes in oxidative phosphorylation, TCA cycle, and glycolysis, which result in lower ATP production [24]. Additionally, decreased NAD levels affect PARPs and sirtuins and lead to the inactivation of downstream molecular pathways, including DNA repair, cellular stress responses, and energy metabolism regulation [5]. Thus, preventing the decline of NAD is suggested as a promising strategy to combat metabolic disorders. Dietary intervention is an ideal way to increase NAD levels in cells and tissues. However, NAD is considered impermeable to the plasma membrane, and NAD administration cannot efficiently increase NAD levels [25]. Therefore, NAD precursors, such as nicotinamide (NAM), nicotinic acid (NA), tryptophan, nicotinamide mononucleotide (NMN), and nicotinamide riboside (NR), are utilized to increase NAD levels in rodents and humans [26]. In particular, NMN and NR administration efficiently boost NAD levels and has beneficial effects for obesity and glucose tolerance in mice [8, 22, 27, 28]. NAM also prevents hepatic steatosis and improves glucose tolerance by reducing oxidative stress and inflammation in diet-induced obese mice [29]. NA improves glucose tolerance and lipid metabolism, and it has already been applied for the treatment of dyslipidemia in humans [30]. In this review, the association of NAD with each metabolic disease and the therapeutic potential of NAD precursors for these diseases are discussed.

**Table 1:** Changes of NAD levels in metabolic tissues with obesity or aging

Model	Tissue	Change	Description	Confirmatiom	References
Obesity	Liver	↓	C57BL/6 congenic mice fed a HFD for 6–8 months	HPLC	[8]
		↓	BALB/c mice fed a HFD for 16–20 weeks	Enzymatic	[96]
		↓	C57BL/6 J mice fed a HFD for 12 weeks	LC/MS	[9]
		↓	C57BL/6 J mice fed a HFHSD for 9 or 18 weeks	LC/MS	[10]
		→	C57BL/6JBomTac mice fed a HFD for 6–48 weeks	LC/MS	[11]
	Skeletal muscle	↓	C57BL/6 mice fed a HFD for 6–8 months	HPLC	[8]
		↓	C57BL/6 mice fed a HFD from 3 to 9 months	HPLC	[12]
		↓	C57BL/6 mice fed a HFD from 6 to 16 weeks	LC/MS	[13]
	Adipose tissue	↓	C57BL/6 mice fed a HFD for 6–8 months	HPLC	[8]
		↓	C57BL/6 congenic mice fed a HFD from 6 to 16 weeks	Enzymatic	[14]
	Hypothalamus	↓	C57BL/6 mice fed a HFHSD for 4 weeks	LC/MS	[15]
		↓	db/db mice at 8 months of age	LC/MS	[15]
Aging	Liver	→	C57BL/6 mice (25–31 months old v.s. 3–6 months old)	HPLC	[8]
		↓	C57BL/6 J mice (24 months old v.s. 6 months old)	HPLC	[16]
		↓	Human (> 60 years old v.s. < 45 years old)	Enzymatic	[17]
		↓	Male C57BL/6 J mice (20 months old v.s. 4 months old)	Enzymatic	[17]
		↓	Male C57BL/6 mice (32 months old v.s. 5 months old)	LC/MS	[18]
		↓	Male C57BL/6 N mice (24 months old v.s. 3 months old)	LC/MS	[19]
	Skeletal muscle	↓	C57BL/6 mice (25–31 months old v.s. 3–6 months old)	HPLC	[8]
		↓	C57BL/6 J mice (22 months old v.s. 6 months old)	Enzymatic	[20]
		↓	C57BL/6 J mice (24 months old v.s. 6 months old)	HPLC	[16]
		↓	Male C57BL/6 mice (32 months old v.s. 5 months old)	Enzymatic	[18]
		↓	C57BL/6 mice (24 months old v.s. 4 months old)	HPLC	[21]
		↓	C57BL/6 J mice (22–24 months old v.s. 1 months old)	Enzymatic	[22]
		↓	Male C57BL/6 N mice (24 months old v.s. 3 months old)	LC/MS	[19]
	Adipose tissue	↓	C57BL/6 mice (25–31 months old v.s. 3–6 months old)	HPLC	[8]
		↓	Male C57BL/6 mice (32 months old v.s. 5 months old)	Enzymatic	[18]

*HPLC* High Performance Liquid Chromatography, *LC/MS* Liquid Chromatography-Mass spectrometry

## NAD synthesis and consuming pathways

**Fig. 2:**
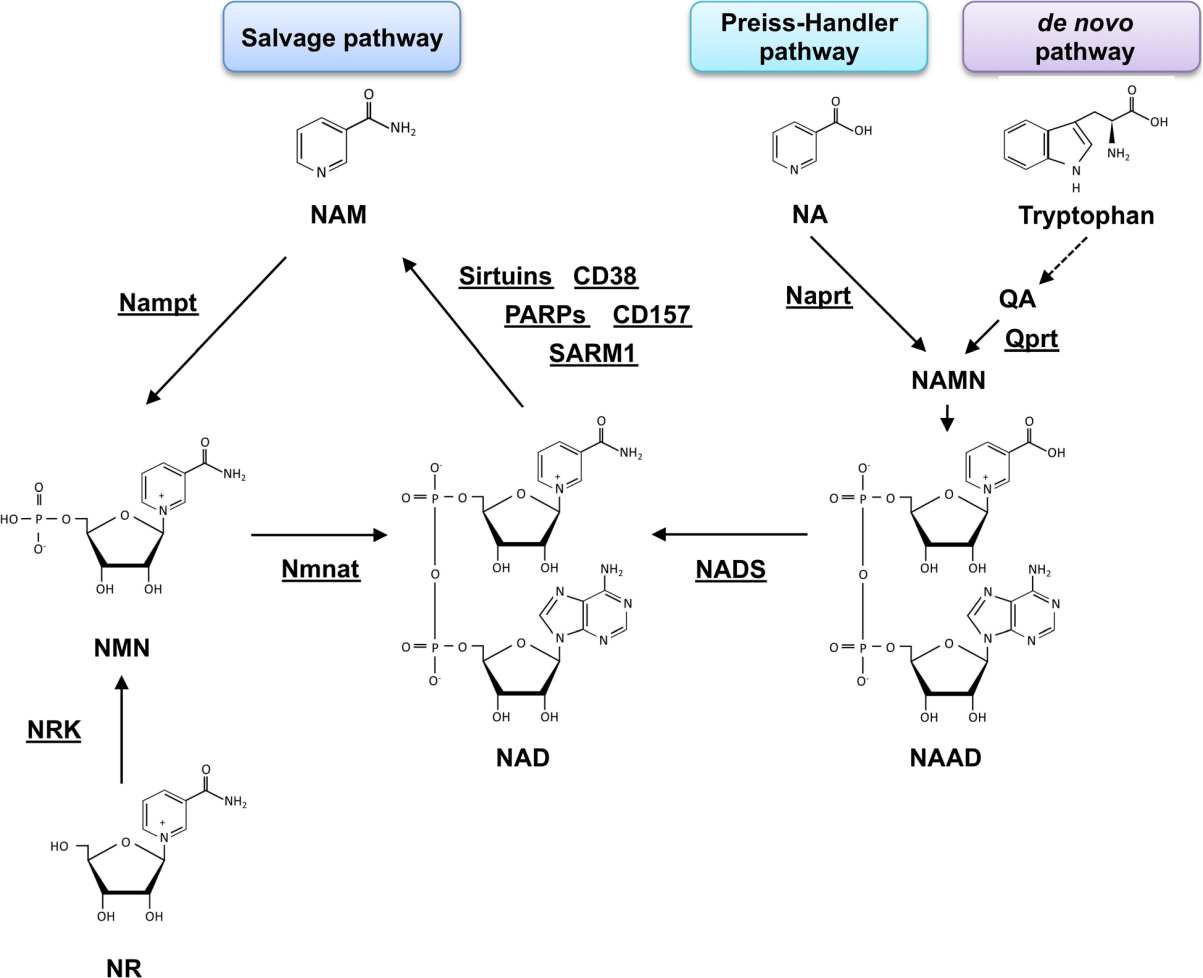
NAD is synthesized through de novo, Preiss-Handler, and salvage pathways. NAM: nicotinamide, NA; nicotinic acid, NAD: nicotinamide adenine dinucleotide, NMN: nicotinamide mononucleotide, NR: nicotinamide riboside, NAAD: nicotinic acid adenine dinucleotide, Nampt: nicotinamide phophoribosyltransferase, Nmnat: NMN adenylyltransferase, NADS: NAD synthase, NRK: nicotinamide riboside kinase

There are three NAD synthesis pathways named salvage, de novo, and Preiss-Handler, where NAD is synthesized from NAM, tryptophan, and NA, respectively (Fig. 2) [31]. These NAD precursors are ingested from dietary sources, and their shortage causes pellagra with characteristic symptoms of inflamed skin, diarrhea, dementia, and sores in the mouth [32]. In mammalian cells, NAD is predominantly synthesized through the salvage pathway where nicotinamide phophoribosyltransferase (Nampt) generates NMN from NAM and 5-phosphoribosyl-1-pyrophosphate (PRPP) [33]. Subsequently, NMN is conjugated to ATP and converted to NAD by NMN adenylyltransferase (Nmnat) [34]. In mammals, there are three Nmnat isozymes that are encoded by different genes. Nmnat1, Nmnat2, and Nmnat3 exist in nucleus, Golgi apparatus, and mitochondria, respectively [34]. The salvage pathway is coupled with NAD-consuming enzymes, such as PARPs, sirtuins, CD38 (T10), CD157 (BST1), and SARM1. These enzymes degrade NAD and generate NAM as a by-product [35, 36]. Nampt is a rate-limiting enzyme in the salvage pathway, and the global deletion of Nampt in mice results in embryonic lethality [33, 37]. Furthermore, the tissue-specific deletion of Nampt in murine metabolic tissues, including skeletal muscle, liver, and adipose tissues, decreases NAD levels in each organ [21, 38, 39]. Most tryptophan, a precursor for de novo synthesis pathway, is consumed in the liver, which is the only organ that possesses all synthetic enzymes of this pathway [40]. However, deficiency of quinolinate phosphoribosyltransferase (Qprt), a key enzyme in the de novo pathway, has no effect in the NAD levels in murine tissues, including the liver [41]. These results indicate that NAD synthesis in mammalian cells largely depends on the salvage pathway. However, a recent study has demonstrated that the de novo pathway contributes to synthesis and maintenance of NAD levels in the macrophages, particularly during aging and inflammation [42]. Therefore, it is possible that the NAD synthesis pathway can switch between the de novo and salvage pathways under certain stress conditions.

Although Nampt functions as a NAD synthesis enzyme in cells, it is also found in serum. It was originally reported as a cytokine named pre-B-cell colony-enhancing factor (PBEF) as well as visfatin, a type of adipokine [43, 44]. The extracellular form of Nampt (eNampt) is secreted from several kinds of cells, including mature adipocytes, pancreatic β-cells, myocytes, and hepatocytes [37, 45, 46]. Reportedly, the intracellular form of Nampt (iNampt) is acetylated in the cytoplasm during normal nutrient status. However, once food becomes scarce, iNampt is deacetylated by SIRT1 [47]. In addition, the deacetylation of Nampt enhances its secretion and enzymatic activity [47]. Interestingly, genetic deletion of Nampt in the adipocytes decreases hypothalamic NAD levels [47]. Likewise, eNampt depletion by neutralizing antibodies has the same effect on hypothalamic NAD levels [47]. These results suggest that eNampt may generate NMN in the blood, thus supplying NMN to various tissues, including the hypothalamus. However, another study determined that eNampt did not participate in the generation of extracellular NMN because the physiological concentrations of NAM, ATP, and PRPP in the plasma were insufficient for the catalysis of Nampt [48]. Therefore, the contribution of eNampt to the generation of extracellular NMN is still under debate.

NR is an alternative NAD precursor, and a study using various chemical inhibitors suggested that NR is incorporated into cells using equilibrative nucleoside transporters (ENTs) [49, 50]. Inside the cells, NR is converted to NMN by nicotinamide riboside kinase (NRK), and knockdown of NRK1 in mammalian cells eliminated NAD synthesis from NR. Interestingly, NRK1 also regulates NAD synthesis from NMN [51]. In NRK1 knockout mice, administration of NMN failed to increase NAD levels in the liver, kidney, and brown adipose tissue [51]. Furthermore, a study using stable isotope-labeled NR and NMN revealed that NMN is dephosphorylated into NR extracellularly [51]. These results suggest that NMN is incorporated into cells after extracellular conversion to NR. Meanwhile, a recent study identified Slc12a8 as a NMN transporter [52]. This study demonstrated that Slc12a8 directly transports NMN across the plasma membrane, and deletion of Slc12a8 in the hepatocytes largely diminished the incorporation of NMN. Slc12a8 is strongly expressed in the small intestine and may contribute to oral uptake of NMN. Therefore, it is possible that uptake pathways of NMN vary with tissue types. Therefore, further studies are necessary to reveal the mode and kinetics of uptake of NAD precursors specific to each tissue and/or cell.

## Obesity

Obesity is a fundamental pathophysiology for various metabolic diseases, such as diabetes, dyslipidemia, and fatty liver. Several studies have revealed that intracellular NAD levels decreased with obesity in multiple murine tissues, including the adipose tissue, skeletal muscles, liver, and hypothalamus [8, 10, 12, 15]. Further, obesity causes low-grade inflammation, and inflammatory cytokines, such as IL-1β, IL-6, and TNF-α, are induced in various tissues, including adipose tissues, liver, and skeletal muscle [53]. These inflammatory cytokines impair the gene expression of Nampt [8, 54]. In humans, several studies have found reduced Nampt levels in adipose tissue, serum, and liver from obese patients [55–57]. However, conflicting results have been reported by several studies [58–62]. It is considered that eNampt is mainly released from adipose tissue [37, 44]. Therefore, it is possible that the increased amount of adipose tissue in obese patients resulted in the enhancement of eNampt secretion. The adipose tissue-specific overexpression of Nampt in mice also shows significant increase in plasma eNampt levels [47]. Reduced iNampt levels correlate with decreased NAD levels in obese tissues; however, the biological significance of increased eNampt in obesity remains unclear. Thus, further studies are warranted to reveal the role of increased eNampt levels in obese patients.

Conversely, NMN or NR administration can prevent the reduction in NAD levels in diet-induced obese mice (Table 2) [27, 28, 65]. Moreover, NR administration partially suppresses weight gain in mice fed a high-fat diet (HFD) by enhancing energy expenditure [8, 28]. Mice with long-term NMN administration exhibit both higher energy expenditure and physical activity, and weight gain during aging is suppressed [27]. Thus, administration of NAD precursors can ameliorate diet- and age-associated weight gain, and nutritional intervention using NMN and NR may be a promising strategy against obesity.

**Table 2:** Therapeutic effects of NAD precursors in metabolic diseases

Model	Administrated NAD precurser	NAD levels in tissues	Metabolic Effects	References
Obesity	NMN (500 mg/kg)	Long-term: Liver ↑, Skeletal muscle↑, WAT → Short term: Liver ↑	Improved glucose tolerance and insulin sensitivity	[8]
	NMN (500 mg/kg)	not shown	Improved insulin secretion and inhibited inflammation	[63]
	NMN 500 mg/kg	Liver↑, Skeletal muscle↑	Improved glucose tolerance, liver citrate synthase activity, and triglyceride accumulation	[64]
	NR (400 mg/kg)	Liver↑, Skeletal muscle↑, BAT↑, WAT→, Brain→	Enhanced mitochondiral biogenesis, Improved insulin sensitivity, and suppressed body weight gain	[28]
	NR (3 g/kg)	Liver ↑	Improved glucose homeostasis and hepatic steatosis, suppressed body weight gain, and protective against diabetic neuropathy	[10]
	NR (400 mg/kg)	Liver (whole) ↑, Liver (mitochondria) ↑,	Improved glucose tolerance, insulin sensitivity, hepatic steatosis, and suppressed body weight gain	[9]
	NR (200 mg/kg)	not shown	Reduced lipid accumulation and fibrosis in liver	[17]
	NR (5-900 ppm)	Liver →	Improved metabolic flexibility	[65]
	NAM (37.5 g/kg or 75 g/kg)	Liver →	Improved glucose tolerance and prevented hepatosteatosis	[29]
Aging	NMN (500 mg/kg)	not shown	Improved lipid profile, glucose tolerance and insulin secretion	[8]
	NMN (100, 300 mg/kg)	Liver↑, Skeletal muscle↑	Inhibited age-induced weight gain, improved insulin sensitivity and plasma lipids, and increased physical activity, energy expenditure, and muscle mitochondrial function	[27]

*WAT* white adipose tissue, *BAT* brown adipose tissue

## Diabetes

### Nampt and insulin secretion

Insulin resistance and subsequent impaired insulin secretion compose the pathophysiology of type 2 diabetes. Both insulin sensitivity and secretion are coordinated by NAD metabolism [26]. Reportedly, NAD levels of islet cells are decreased in heterozygous whole body Nampt knockout mice, and glucose-stimulated insulin secretion (GSIS) is compromised in these mice [37]. Conversely, NMN administration recovers NAD, and ameliorates impaired GSIS in these mice [37]. Although eNampt was reported as a ligand for the insulin receptor (IR) and had an insulin-mimetic effect, the study has been retracted [44]. Later studies also argue that eNampt does not directly activate the insulin-signaling pathway in β-cell lines [37]. However, several studies have suggested positive effects of eNampt on insulin secretion [37, 63, 66]. Reportedly, mice fed a fructose-rich diet (FRD) show significantly reduced eNampt levels, leading to increased islet inflammation and impaired insulin secretion [63]. Islet cells in FRD-fed mice exhibited increased expression of inflammatory cytokines, including TNFα and IL-1β, whereas NMN administration reduced IL-1β expression and restored the decreased insulin secretion in FRD-fed mice, suggesting that eNampt regulates β-cell function through a mechanism of NAD synthesis [63].

### Adipocyte Nampt and insulin resistance

Adipocyte-specific deletion of Nampt caused insulin resistance, and this effect is systemic and not restricted to the adipose tissue [67]. Loss of Nampt in adipocytes increases CDK5 and PPARγ phosphorylation, leading to reduce the serum adiponectin levels and conversely increase serum free fatty acid levels [67]. Thus, adipocyte-specific Nampt knockout (FANKO) mice have demonstrated a systemic insulin resistance when fed a normal chow diet. A recent study has demonstrated that FANKO mice are resistant to obesity induced by HFD and lack healthy adipose tissue expansion [68]. Although adipose tissue mitochondria in HFD-fed FANKO mice have a reduced respiratory capacity, the mice exhibit improved glucose tolerance compared with control mice [68]. Furthermore, FANKO mice exhibit reduced food intake [68]. These results suggest that Nampt in adipocytes is necessary for healthy expansion during diet-induced obesity and it is also important for the maintenance of insulin sensitivity in normal nutrient status. Thus, the roles of Nampt in adipose tissues may differ by the nutrient status.

### Skeletal muscle Nampt and metabolic disorders

The role of skeletal muscle Nampt in metabolic disorders has been reported using muscle-specific Nampt-overexpressing mice [12, 21, 69]. Although Nampt-overexpressing mice have higher NAD levels in skeletal muscles, there is no significant difference in weight gain between Nampt-overexpressing and control mice fed an NCD or HFD [12, 69]. Nampt-overexpressing mice fed a very HFD are partially protected against body weight gain but not against diet-induced insulin resistance [69]. However, Nampt-overexpressing mice have higher exercise endurance capacity and enhanced mitochondrial gene expression [69]. Furthermore, muscle-specific Nampt knockout mice display progressive muscle degeneration with a significant reduction in NAD levels in muscle [21]. Respiratory capacity also decreases in mitochondria from muscle-specific Nampt knockout mice [21]. It is of further interest to investigate the effect of skeletal muscle-specific deletion of Nampt against glucose tolerance.

### Nmnat3 and insulin resistance

Recently, the authors reported that systemic overexpression of Nmnat3 in mice can efficiently increase NAD levels in various tissues and ameliorate the onset of diet- and age-associated insulin resistance [13]. In the skeletal muscles of Nmnat3-overexpressing (Nmnat3 Tg) mice, the increase in TCA cycle intermediates was accompanied by repletion of mitochondrial NAD level, suggesting the activation of the TCA cycle. Additionally, the fuel for energy metabolism was shifted from carbohydrates to fatty acids. Furthermore, overexpression of Nmnat3 modulates the ratio of mitochondrial respiratory chain complexes, which might be associated with lower reactive oxygen species (ROS) generation during aging [13]. Of note, Nmnat3 Tg mice have significantly increased concentrations of nicotinamide guanine dinucleotide (NGD), a NAD analog [13]. However, it is still unclear whether increased NGD levels contribute to the phenotypes in Nmnat3 Tg mice, and further studies are awaited.

### CD38 and insulin resistance

A recent study has demonstrated that the decline of NAD levels with aging is largely dependent on CD38 [18]. CD38 has an enzymatic activity catalyzing the degradation of NAD into NAM and ADP-ribose (ADPR). CD38 also has ADPR cyclase activity generating cyclic-ADPR from NAD [70]. Interestingly, the knockout of CD38 increases basal NAD levels in tissue, suggesting the importance of NAD degradation by CD38 in regulating NAD levels. Additionally, expression of CD38 in various tissues is remarkably elevated with aging, and NAD consumption is also accelerated in correlation with CD38 levels. In contrast, CD38 deficiency in mice eliminates NAD decline during aging [18]. Although CD38 has been found in plasma membrane as an ectoenzyme, it has been also detected in mitochondria [71, 72]. Importantly, CD38-deficient mice display improved age-associated glucose intolerance. This benefit should be attributed to the upregulation of mitochondrial function by increasing mitochondrial NAD levels and SIRT3 activity [18]. Recently, a CD38 specific inhibitor, 78c, has been reported to have beneficial effects against age-associated physical declines including glucose tolerance and exercise capacity [73]. Treatment with 78c prevents NAD decline with aging and activates sirtuins, AMPK, and PARPs.

### NAD precursors and diabetes

Several research groups used the precursors of NAD, NR or NMN, to increase the level of NAD and showed they could improve insulin resistance due to obesity [8, 28, 64]. The effect of NAD precursors is mainly through the enhancement of sirtuin pathways. The intracellular level of NAD is increased in liver after the administration of NR, leading to activated SIRT1 and SIRT3 [28]. Then SIRT1 promotes the deacetylation of FOXO1, inducing SOD2. SIRT3 promotes the deacetylation of both SOD2 and NDUFA9 [28]. In addition, administered NR contributes to the enrichment of the mitochondrial content in skeletal muscle and brown adipose tissues. Thus, the administration of NR protects mice against obesity and glucose tolerance, increasing fatty acid oxidation and energy expenditure and improving insulin sensitivity [28]. As well as NR, the administration of NMN increases hepatic NAD level and ameliorates the insulin sensitivity in liver. Besides, NMN relieves oxidative stress and the inflammatory response which is induced by diet-induced-obesity and recovers the perturbed circadian rhythm [8]. Long-term NMN administration also ameliorates age-associated insulin resistance and prevents changes in gene expression with aging [27]. Aged mice administered NMN also maintained better mitochondrial respiratory capacity in skeletal muscle, which may contribute to improved glucose tolerance.

Recently, a research group examined the effect of long-term administration of NAM in mice. [29]. Although the level of NAD and the mean or maximum lifespan were unchanged, the administration of NAM restored some aging-related metabolic decline including increased protein carbonylation and the reduction of oxygen consumption rates. Consequently, NAM administration ameliorates glucose tolerance during diet-induced obesity. Thus, NAM promotes a healthy lifespan without obvious adverse effects, and this can be translated into humans. Previous studies have also demonstrated that NAM administration ameliorates sustained hyperglycemia by increasing β-cell proliferation in various diabetic rodent models [74, 75]. In particular, NAM can rescue streptozotocin (STZ)-induced β-cell damage and diabetes (model of type 1 diabetes [T1DM]) [75]. Thus, oral NAM administration is thought to be a therapeutic agent for T1DM. Although small-scale clinical trials have reported the beneficial effects of NAM against T1DM [76], a large-scale randomized controlled trial demonstrated that NAM intervention in patients with confirmed anti-islet cell antibodies failed to prevent the onset of T1DM [77]. For T1DM treatment, NMN and NR are still attractive candidates because NAD-mediated SIRT1 activation augments GSIS in β-cells [78]. Further studies are needed to investigate the effects of NMN or NR in the prevention and/or treatment of T1DM.

## Dyslipidemia

Dyslipidemia is caused by both nutritional and genetic factors, and it is associated with various metabolic disorders and cardiovascular diseases. NA was the first therapeutic agent to treat dyslipidemia and has been used to prevent cardiovascular disease clinically for a long time [79]. NA lowers the level of triglycerides and low-density lipoprotein cholesterol (LDL-C) and raises the level of high-density lipoprotein cholesterol (HDL-C) [79]. However, the mechanisms of how NA improves dyslipidemia remain unclear. Several studies have indicated that these effects are due to the activation of the G protein-coupled receptor GPR109A in adipocytes [80–82]. Alternatively, a study using Gpr109A-deficient mice and the clinical trials for GPR109 agonists contradict this hypothesis [83]. A recent study demonstrated that NA administration increases NAD levels, and the subsequent activation of sirtuins contributes to improved lipid metabolism [84]. Therefore, NMN and NR, which activate SIRT1 without activating GPR109A, can be potential therapeutic options [28]. Although the beneficial outcome of NA on dyslipidemia has been confirmed by numerous studies, two recent large clinical trials concluded that NA combined with statin therapy did not provide additional benefits over statin monotherapy against cardiovascular incidents [85, 86]. However, the favorable effect of NA on LDL-C, triglycerides, and HDL-C has been clearly shown, and NA administration is still used as an adjuvant therapy to reduce atherogenic lipoprotein burden.

## NAFLD and hepatic steatosis

Excess calorie intake causes ectopic lipid accumulation in the liver, known as non-alcoholic fatty liver disease (NAFLD) [87]. The progression of NAFLD leads to hepatic steatosis, hepatitis, liver cirrhosis, and ultimately, liver dysfunction. Further, these hepatic diseases occasionally coincide with hepatocellular carcinoma [87]. In NAFLD, the ectopic lipid accumulation results in increased ROS and the mitochondrial dysfunction [88]. It is reported that NAD levels are decreased in the liver of the diet-induced NAFLD mice model [89]. Along with this, the activity of both SIRT1 and SIRT3 is decreased [89–92]. Conversely, SIRT1 overexpression restores the diet-induced hepatic steatosis [93, 94]. NR administration also protects against mitochondrial dysfunction with diet-induced NAFLD through NAD elevation and subsequent SIRT1 activation [9, 28]. In humans, patients with NAFLD present lowered Nampt levels in the liver [55]. Altogether, the administration of NAD precursors is considered a potential therapeutic option for the treatment of NAFLD. In contrast, the inhibition of Nampt using FK866 promotes lipid accumulation and hepatic steatosis in HFD-fed mice [95]. FK866 treatment decreases protein levels of SIRT1 and phospho-AMPK, and also increases the gene expression of SREBP1 and fatty acid synthase in the liver of HFD-fed mice [95]. Similarly, dominant-negative Nampt-overexpressing (DN-Nampt Tg) mice display NAFLD-like phenotypes, including lipid accumulation, chronic inflammation and impaired insulin sensitivity, in the liver. NR administration to DN-Nampt Tg mice can rescue the NAFLD-like phenotypes [17]. miR-34a negatively regulates the expression of Nampt and SIRT1 during obesity [96]. Obesity induces the expression of miR-34a, which resulted in the reduction of Nampt levels and subsequent aggravation of hepatic lipid accumulation in vivo. In contrast, reducing miR-34a levels in obese mice restores Nampt and NAD levels and improves inflammation, glucose intolerance, and hepatic steatosis through the Nampt-NAD-SIRT1 axis [96]. Thus, it has been proposed that the Nampt/NAD/SIRT1 axis can suppress hepatic steatosis in HFD-fed mice.

## Human clinical trials

In various mouse models of human disease, the benefit of NAD precursors, in particularly NMN and NR, has been demonstrated (Table 2). Currently, there are several ongoing human clinical trials or recently reported trials (Table 3). The first report of oral NR administration revealed that NR could increase NAD levels in plasma and peripheral blood mononuclear cells (PBMC) [97]. In this study, consented healthy volunteers received a single dose of 100, 300, and 1000 mg NR in different sequences with 7-day washout periods between data collection. Two participants reported flushing at the dose of 300 mg, but no other serious adverse side effects were reported. Interestingly, NR administration also increased nicotinic acid adenine dinucleotide (NAAD) levels in PBMC [97]. Another clinical trial of oral NR administration for 8 days was conducted as an open-label, non-randomized study in healthy volunteers [98]. In this study, participants took gradually incremented doses of NR from 250 mg to 2000 mg per day, and NR administration was well tolerated with no unfavorable side effects [98]. Importantly, NR administration in healthy subjects significantly increased plasma NAD levels in correlation with plasma NR levels [97, 98]. Chronic NR administration to healthy-aged volunteers (average age, 65 years) was reported [99]. In this study, participants were orally administered 500 mg NR twice daily for 6 weeks, and there were no serious side effects. NAD levels in the NR treatment group were significantly increased in PBMC by approximately 60% compared with that in the placebo group. Consistent with previous reports, NAAD levels were also significantly increased in the NR treatment group [99]. This study also reported that NR treatment lowered systolic blood pressure and arterial stiffness. Similarly, acute NR supplementation in old individuals increased NADH and NADPH levels and improved exercise performance [100]. Another clinical trial in obese men investigated safety and insulin sensitivity [101]. Men with a body mass index > 30 kg/m^2^, with an age range of 40–70 years, were randomly assigned to 12 weeks of NR administration (1000 mg twice daily) or placebo. Although no serious adverse events occurred with NR administration, insulin sensitivity, endogenous glucose production, and glucose disposal and oxidation were not improved [101]. Another clinical trial with a combination of NR and pterostilbene (NRPT), a polyphenol found in blueberries, studied healthy volunteer subjects [102]. NR and NPRT at recommended dose (NRPT 1X; 250 mg of NR plus 50 mg of PT), and NRPT at double dose (NRPT 2X; 500 mg of NR plus 100 mg of PT) were orally administrated to participants for 8 weeks. In this study, NAD levels were increased in a dose-dependent manner (approximately 40% in NRPT 1X and 90% in NRPT 2X), and no serious adverse side effects were observed [102]. Clinical trials to examine the safety and pharmacokinetics of NMN have been recently initiated in the United States and Japan [103], and the results of these trials are yet to be seen.

**Table 3:** Human clinical trials of NAD precursors, NMN and NR

Molecule	Aim	Design	Intervention	Outcome	References (Clinical trials.gov or UMIN-CTR identifier)
NR	Study for PK and safety in healty volunteer.	Randomized, double-blind, crossover study. Healthy volunteers (*n* = 12), Age from 33 to 55, BMI from 18.5 to 29.9	Oral administration. Single administration at 100, 300, and 1000 mg	Increased NAD and NAAD levels in PBMC. No seriouse adverse side effects. Two individuals self-reported flushing at the 300 mg dose.	[97] (NCT02191462)
NR	Study for PK and safety in healty volunteer.	Non-randomized, open-label, non-placebo controlled study. Healthy volunteers (*n* = 8), Age from 21 to 50	Oral administration. Dose-escalation at 250 mg (Day 1, 2), 500 mg (Day 3, 4), 1000 mg (Day 5, 6), and 2000 mg (Day 7, 8)	Approximately 100% Increase in NAD level in whole blood with a potisitve colleration of NR level. No seriouse adverse side effects.	[98] (NCT02689882)
NR	Study for safety and efficacy against physical activities in elderly people.	Non-randomized, open-label, crossover study. Healthy volunteers (*n* = 30), Age from 55 to 79, BMI =24 ± 4	Oral administration. Crossover of placebo for 6 weeks and NR 500 mg twice daily for 6 weeks	Increased NAD and NAAD levels in PBMC. Well torelated and no seriouse adverse side effects. Lowered systolic blood pressure and arterial stiffness in NR treated group.	[99] (NCT02921659)
NR	Study for kinetics and efficacy against exercise performance in elderly people.	Randomized, double-blind, crossover study. Healthy young (*n* = 12, 22.9 ± 1.0 years) and elderly (n = 12, 71.5 ± 1.0 years) volunteers	Single oral administration. Crossover of placebo and NR 500 mg	Increased NADH and NADPH levels in RBCs. Regarding exercise performance, Isometric peak torque and fatigue index is improved in NR treated old group.	[100] N/A
NR	Study for safety and efficacy against insulin sensitivity in obese men	Randomized, placebo-controlled, double-blinded study. Healthy, sedentary, obese men (*n* = 40), Age from 40 to 70, BMI > 30	Oral administration. Placebo or NR 1000 mg twice daily for 12 weeks.	Increased urinary NR, NAM, MeNAM in NR treasted group. Insulin sensitivity, endogenous glucose production, and glucose disposal and oxidation were not improved Resting energy expenditure or body composition was not affected. No serious adverse side effects.	[101] (NCT02303483)
NRPT (NR and pterostilbene)	Study for safety and efficacy against NAD sustainability in elderly people	Randomized, placebo-controlled, double-blinded study. Healthy volunteers (*n* = 120), Age from 60 to 80	Oral administration. Placebo, 1XNRPT (250 mg NR and 50 mg PT), or 2XNRPT (500 mg NR and 100 mg PT) daily for 8 weeks.	Increased NAD level in whole blood with a dose-dependent manner. Total and LDL cholesterol levels were increased in NPRT treated group. No lserious adverse side effects.	[102] (NCT02678611)
NMN	Study for efficacy against in insulin sensitivity and β-cell functions in female elderly people.	Randomized, placebo-controlled, double-blinded study. Postmenopausal women 55–75 years old, BMI 25.0–44.9, and pre-diabetic.	Oral administration. Placebo or NMN 250 mg daily for 8 weeks.	N/A	(NCT03151239)
NMN	Study for PK and safety in healty volunteer.	Non-randomized, open-label, non-placebo controlled study. Male healthy volunteers (*n* = 10), Age from 40 to 60	Oral administration. Single administration. Dose is not described.	N/A	[103] (UMIN000021309)
NMN	Study for PK, safety, and effects on hormones in healty volunteer.	Randomized, dose comparison, double-blinded study. Healthy volunteers (*n* = 20), Age from 50 to 70	Oral administration. Single administration in dose of 100 mg or 200 mg of NMN.	N/A	(UMIN000025739)
NMN	Study for PK and safety in healty volunteer.	Non-randomized, open-label, non-placebo controlled study. Male healthy volunteers (*n* = 10), Age from 40 to 60	Oral administration. Long-term NMN administration for 8 weeks. Dose is not described.	N/A	[104] (UMIN000030609)

*PK* pharmacokinetic, *BMI* body mass index, *LDL* low density lipoprotein

## Conclusions

NAD metabolism is spotlighted as a therapeutic target for metabolic disorders, such as obesity, diabetes, dyslipidemia, and fatty liver. The genetic manipulation of NAD synthesis or catabolizing enzymes has established that reduction in NAD levels causes metabolic disorders in mice. Furthermore, mounting evidence has demonstrated that complementing NAD with NAD precursors ameliorates various metabolic diseases. Recently, several human clinical trials have been reported. Overall, NR administration is safe, well tolerated, and can efficiently increase NAD levels in healthy volunteers. However, efficacy in patients with metabolic disorders remains unclear, and further studies are awaited. Moreover, some small molecules boosting NAD levels have been reported [73, 104, 105]. Outcomes of these molecules against metabolic diseases in patients should be clarified in future studies. It is also demonstrated that NMN and NR are contained in natural foods, including cow milk, broccoli, cucumber, avocado, and beef [27, 106, 107]. Thus, NAD metabolism is considered a practical target for a nutritional intervention.
